# Arterial thromboembolism in multiple myeloma in the context of modern anti-myeloma therapy

**DOI:** 10.1038/s41408-021-00513-4

**Published:** 2021-06-25

**Authors:** Rajshekhar Chakraborty, Lisa Rybicki, Jason Valent, Alex V. Mejia Garcia, Beth M. Faiman, Jack Khouri, Christy J. Samaras, Faiz Anwer, Alok A. Khorana

**Affiliations:** 1 Multiple Myeloma and Amyloidosis Program, Herbert Irving Comprehensive Cancer Center, Columbia University Medical Center, New York, NY USA; 2 Blood and Marrow Transplant Program, Taussig Cancer Institute, Cleveland Clinic, Cleveland, OH USA; 3 Department of Hematology and Medical Oncology, Taussig Cancer Institute, Cleveland Clinic, Cleveland, OH USA

**Keywords:** Myeloma, Risk factors

Dear Editor,

Although the incidence and risk factors of venous thromboembolism [VTE] is well characterized in multiple myeloma [MM], little is known regarding the characterization of arterial thromboembolism [ATE] in the context of modern anti-myeloma therapy. Since MM is a cancer of older adults with several shared risk factors for cancer and cardiovascular disease, ATE remains an area of major concern. Notably, population-based studies from Sweden have demonstrated an increased risk of ATE in MM compared to matched controls, with an incidence of 3.8% in the first year [1]. Another study by the HOVON Group reported a high ATE incidence of 5.6% among newly diagnosed transplant-eligible MM patients receiving doxorubicin-based regimens [2]. However, data from older literature captured ATE incidence prior to the introduction of proteasome inhibitors and immunomodulatory drugs (IMiDs). The objective of our retrospective cohort study was to investigate the incidence, risk factors, and nature of ATE events in newly diagnosed MM in the context of modern anti-myeloma therapy.

We included all consecutive MM patients treated at the Cleveland Clinic from 1/1/2008 to 12/31/2018. We identified potential baseline disease-related, patient-related and treatment-related risk factors a priori and extracted from medical records. The primary endpoint was to estimate ATE incidence in the first year after treatment initiation. Secondary endpoints were to identify risk factors for ATE and association of ATE with overall survival [OS].

We calculated the cumulative incidence of ATE by 12 months. Death without ATE was a competing risk for ATE. Fine and Gray regression was used to identify univariate risk factors for ATE and results were reported as hazard ratio [HR] with 95% confidence intervals [CI]. We tested the following pre-treatment variables: year of treatment, age, sex, race, immunoglobulin subtype, International Staging System [ISS] stage, bone marrow plasma cell percentage, M-protein, involved/uninvolved free light chain ratio, karyotype, high-risk FISH, lactate dehydrogenase, creatinine, calcium, hemoglobin, prior ATE, prior VTE, body mass index, diabetes mellitus, chronic kidney disease, hypertension, hyperlipidemia, total leukocyte count, platelet count, liver disease, acute infection (within 90 days before treatment), erythropoietin use, clotting disorders, autoimmune disease, hyperviscosity, dexamethasone dose, doxorubicin use, multi-agent chemotherapy, smoking status, IMiD use, and concurrent anti-platelet/anti-thrombotic therapy. If HR could not be calculated, Gray test was used instead to assess association with ATE; this occurs for variables with categories that have 0% or 100% ATE events. Survival based on occurrence of ATE was assessed by landmark analyses at 6 and 12 months. For this analysis, Kaplan–Meier survival estimates at 5 years were calculated for those with or without ATE by the landmark along with log-rank test *p* value.

A total of 1029 consecutive patients met inclusion criteria, of whom 934 patients with available data on initial anti-myeloma therapy were analyzed. The baseline clinical and demographic characteristics are summarized in Table 1. The median age at treatment initiation was 63 years [range, 22–94]. Approximately one-fifth of patients were Black and more than half were male. The cohort was roughly equally divided into ISS stages I, II, and III disease, with one-fourth of patients having high-risk FISH cytogenetics at diagnosis. ATE before MM diagnosis was present in 12% of patients. Approximately one-fifth were actively smoking or had quit within 10 years prior to MM diagnosis. A 3-drug induction regimen with VRD (bortezomib-lenalidomide-dexamethasone) or VCD (bortezomib-cyclophosphamide-dexamethasone) was initiated in 52% of patients. Less than 6% had received high-dose dexamethasone [>16o mg per cycle]. The following anti-platelet/anti-thrombotic agents were administered: none [33%], low-dose aspirin [55%], prophylactic low molecular weight heparin [LMWH] (4%), and warfarin or therapeutic LMWH [7%].

**Table 1:** Baseline clinical and demographic characteristics.

Variable [Number of Patients with Available Data]	Patients [934]	
	*N*	%
Male sex [934]	516	55.2
Race [925]:		
White	738	79.8
Black	175	18.9
Other	12	1.3
Age at treatment initiation[934]	Median: 63 years [22–94]	NA
Multiple Myeloma Subtype [934]:		
IgG	477	51.1
IgA	209	22.4
IgM	11	1.2
Others	237	25.4
Albumin [g/dl] [817]	Median: 3.7 [1.1–5.2]	NA
Β-2 microglobulin [mcg/ml] [762]	Median: 3.8 [0.2–78.0]	
ISS stage [808]		
I	267	33.0
II	260	32.2
III	281	34.8
Percentage BMPCs [866]	50 [1–100]	NA
Serum M-protein [g/dl] [822]	Median: 1.95 [0–10.49]	NA
Involved/Uninvolved sFLC ratio [743]	80 [0.5–64,775]	NA
Abnormal Metaphase Cytogenetics [781]	146	18.7
High-Risk FISH Cytogenetics^a^ [604]	143	23.7
LDH > ULN [643]	178	27.7
History of ATE [934]	114	12.2
History of VTE [931]	60	6.4
BMI [799]		
<25	208	26
25–29.9	291	36.4
30–34.9	195	24.4
35–39.9	66	8.3
≥40	39	4.9
History of Cardiac Disease^b^ [933]	164	17.6
History of DM [934]	157	16.8
History of CKD [931]	106	11.4
History of HTN [932]	464	49.8
History of HLD [923]	288	31.2
Acute Infection at Diagnosis^c^ [932]	37	4.0
History of Autoimmune Disease [925]	60	6.5
Smoking History [922]^d^		
Never	523	56.7
Former	222	24.1
Current	177	19.2
Initial Treatment Regimen [934]:		
VRD	378	40.5
VD	204	21.8
RD	179	19.2
VCD	104	11.1
Others	69	7.4
Dexamethasone Dose per Cycle [900]:		
<120 mg	168	18.7
120–60 mg	681	75.7
>160 mg	51	5.7
Initial Thromboprophylaxis Regimen [863]		
None	288	33.4
ASA	477	55.3
Prophylactic LMWH	35	4.1
Warfarin/Therapeutic LMWH	63	7.3

*ISS* International Staging System, *BMPC* Bone Marrow Plasma Cells, *NA* Not Applicable, *sFLC* Serum Free Light Chain, *FISH* Fluorescence in situ Hybridization, *LDH* Lactate Dehydrogenase, *ULN* Upper Limit of Normal, *ATE* Arterial Thromboembolism, *VTE* Venous Thromboembolism, *BMI* Body Mass Index, *DM* Diabetes Mellitus, *CKD* Chronic Kidney Disease, *HTN* Hypertension, *HLD* Hyperlipidemia, *VRD* Bortezomib-Lenalidomide-Dexamethasone, *VD* Bortezomib-Dexamethasone, *RD* Lenalidomide-Dexamethasone, *VCD* Bortezomib-Cyclophosphamide-Dexamethasone, *ASA* Aspirin, *LMWH* Low Molecular Weight Heparin.

^a^High‐risk FISH abnormality was defined by the presence of deletion(17p), t(4;14), t(14;16), and/or t(14;20).

^b^Cardiac disease was defined as congestive heart failure, coronary artery disease [including acute myocardial infarction], and/or arrhythmia.

^c^Defined as acute infection within 90 days prior to treatment initiation.

^d^Current smokers were defined as patients who were smoking at diagnosis or had quit smoking less than 10 years before diagnosis. Former smokers were defined as patients who had quit smoking more than 10 years before diagnosis.

A total of 25 ATE events were observed within a year of treatment initiation. The cumulative incidence of ATE at 6 and 12 months was 2.0% [95% CI, 1.2–3.0] and 2.7% [95% CI, 1.8–4.0] respectively. The Kaplan–Meier curve for cumulative incidence of ATE, along with that of VTE for comparison, is shown in Fig. 1. The median time to ATE from treatment initiation was 4.8 months [range, 0.2–11.5 mo.]. The nature of ATEs are as follows: acute ischemic stroke [*n* = 10; 40%], acute myocardial infarction [*n* = 10; 40%], transient ischemic attack [*n* = 2; 8%], peripheral artery embolism [*n* = 2; 8%], and superior mesenteric artery thrombosis [*n* = 1; 4%]. Of 15 patients for whom response status at the time of ATE was available, 4 had very good partial response [VGPR], 7 had PR, and 4 had stable disease.

**Fig. 1:**
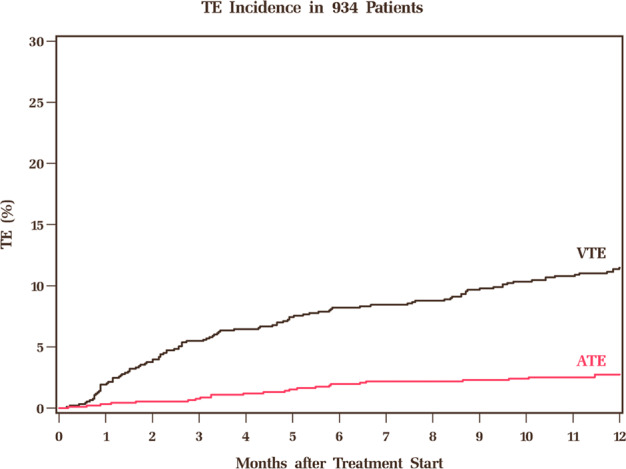
Incidence of arterial and venous thromboembolism in the first year after treatment initiation. ATE Arterial Thromboembolism, VTE Venous Thromboembolism.

On univariate analysis, the following factors were significantly predictive of ATE: ISS stage III disease [vs I/II; HR = 2.62; 95% CI, 1.06–6.51; *p* = 0.038], prior ATE [HR = 6.86; 95% CI, 3.14–15.0; *p* < 0.001], diabetes mellitus [HR = 2.89; 95% CI, 1.28–6.52; *p* = 0.011], chronic kidney disease [HR = 3.58; 95% CI, 1.48–8.69; *p* = 0.005], hyperlipidemia [HR = 2.66; 95% CI, 1.19–5.92; *p* = 0.017], acute infection [HR = 4.92; 95% CI, 1.71–14.2; *p* = 0.003], and current smoker [vs former/never smoker; HR = 2.37; 95% CI, 1.05–5.35; *p* = 0.037]. Of note, use of IMiDs in induction therapy was not associated with an increase in ATE risk [HR = 0.54; 95% CI, 0.24–1.20; *p* = 0.13]. We did not find any significant association between the use of thromboprophylactic agents and incidence of ATE.

Subsequently, we evaluated the association of ATE with OS. Of 25 patients with ATE events, nine were alive with a median follow-up after ATE diagnosis of 23.9 months [range, 7.9-71.8 months]. The 5-year OS among patients with or without ATE at 6-month landmark was 39% and 60% respectively [*p* = 0.004] and that at 12-month landmark was 45% and 63% respectively [*p* < 0.001]. The Kaplan–Meier curves for survival using landmark analysis at 6 and 12 months is shown in the figure in Supplementary Appendix A.

Our study demonstrates that the incidence of ATE in the first year after myeloma diagnosis is 2.7% in the current era, which is substantially lower than prior estimates with cytotoxic therapies. Notably, the high ATE incidence (5.6%) seen in the study by the HOVON group was in the context of doxorubicin-based regimens [2], which could be due to the pro-coagulant effect of doxorubicin on platelets [3]. Our data is in line with the recently published Myeloma XI trial, which showed an ATE incidence of 1.3% and 2.4% in transplant-eligible and transplant-ineligible pathways respectively [4]. Furthermore, data from our study confirms the association of ATE with worsened OS in the current era, consistent with the Swedish myeloma database and Myeloma XI trial [4,5,]. However, we acknowledge, that there may not be a causal relationship between ATE and survival, since ATE is associated with several co-morbidities and a high tumor burden, which may be the drivers of mortality in these patients. The only tumor-specific risk factor that we could identify was ISS stage III, which implies a high tumor burden. Although IMiD use is a well-established risk factor for VTE in MM [6], the association with ATE is less well defined. Due to a low event rate, it remains difficult to observe meaningful difference in ATE incidence between treatment arms in randomized clinical trials [7]. With a large sample size, our study shows that IMiD use may not associated with increased ATE incidence in the first year of treatment. However, prolonged IMiD maintenance therapy beyond first year may be associated with a small increase in ATE risk, as shown in the Myeloma XI trial [4].

Our study has limitations. First, our database did not have incidence of clonal hematopoiesis of indeterminate potential [CHIP], which is a non-modifiable risk-factor for atherosclerotic cardiovascular disease [8]. However, a recent study on 629 transplanted myeloma patients did not demonstrate CHIP as a predictor of ATE in this population at a median follow-up of ~10 years [9]. Second, despite a large sample size, the total number of ATE events was low, hence, multivariable analysis of risk factors could not be performed. Unfortunately, confounding can be present in retrospective analyses; hence, we need studies with more events to identify independent risk factors for ATE. Third, we did not have a matched control group of patients without myeloma. However, based on data from the SEER-Medicare linked database, the 6-month cumulative incidence of ATE in patients with cancer and matched controls is 4.7% and 2.2% respectively, with the highest risk in patients with lung cancer (8.3%) [10]. Hence, the incidence of ATE in patients with MM treated in the modern era was comparable to Medicare enrollees without a cancer diagnosis.

In summary, we show a small but significant risk of ATE in the first year after myeloma diagnosis in the current era, with several modifiable risk factors, including diabetes, hyperlipidemia, and smoking. A history of ATE was present in 12% of patients prior to myeloma diagnosis, which was the strongest risk factor for subsequent ATE. Patients with myeloma should undergo a thorough risk assessment for ATE at diagnosis. There is a paucity of data on primary prophylaxis for ATE in patients with cancer. A phase III RCT comparing LMWH (nadroparin) to placebo for prevention of VTE and ATE in solid tumor patients demonstrated a decrease in ATE risk (stroke and peripheral thrombosis) by 50% (0.4% in nadroparin arm and 0.8% in placebo arm) [11]. However, the primary endpoint included a composite of VTE and ATE and lacked power to detect a difference in ATE specifically. Similarly, a recent update from the CASSINI trial demonstrated a numerically lower incidence of ATE with rivaroxaban compared to placebo (1.0% vs 1.7%), however, was not statistically significant likely due to low number of events [12]. Future studies should assess the role of primary and secondary prophylaxis for ATE, especially in high-risk patients.

## Supplementary information

Figure Legend

Supplementary Appendix A
