# Supplementary material for: Arterial thromboembolism in multiple myeloma in the context of modern anti-myeloma therapy
Source: Blood Cancer J. 2021 Jun 25;11(6):121. doi: 10.1038/s41408-021-00513-4 (PMC8233391; doi:10.1038/s41408-021-00513-4)
Supplement: Supplementary file 2 — Supplementary Appendix A [file 41408_2021_513_MOESM2_ESM.pdf]

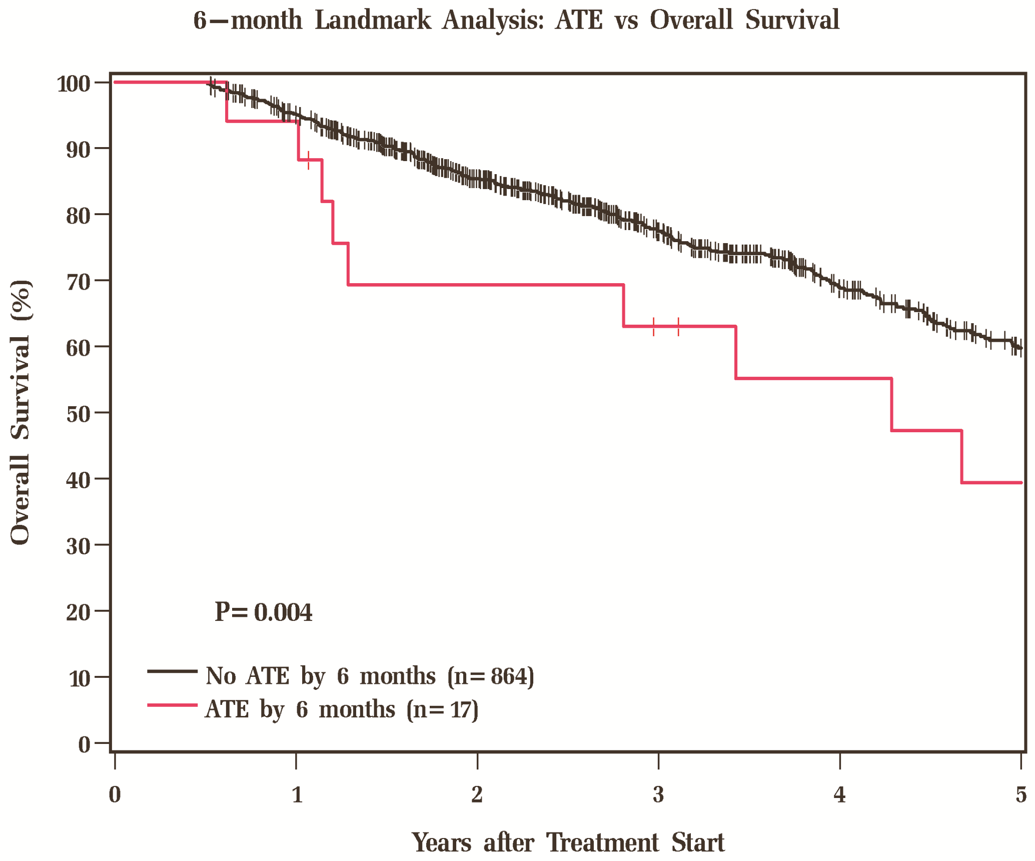

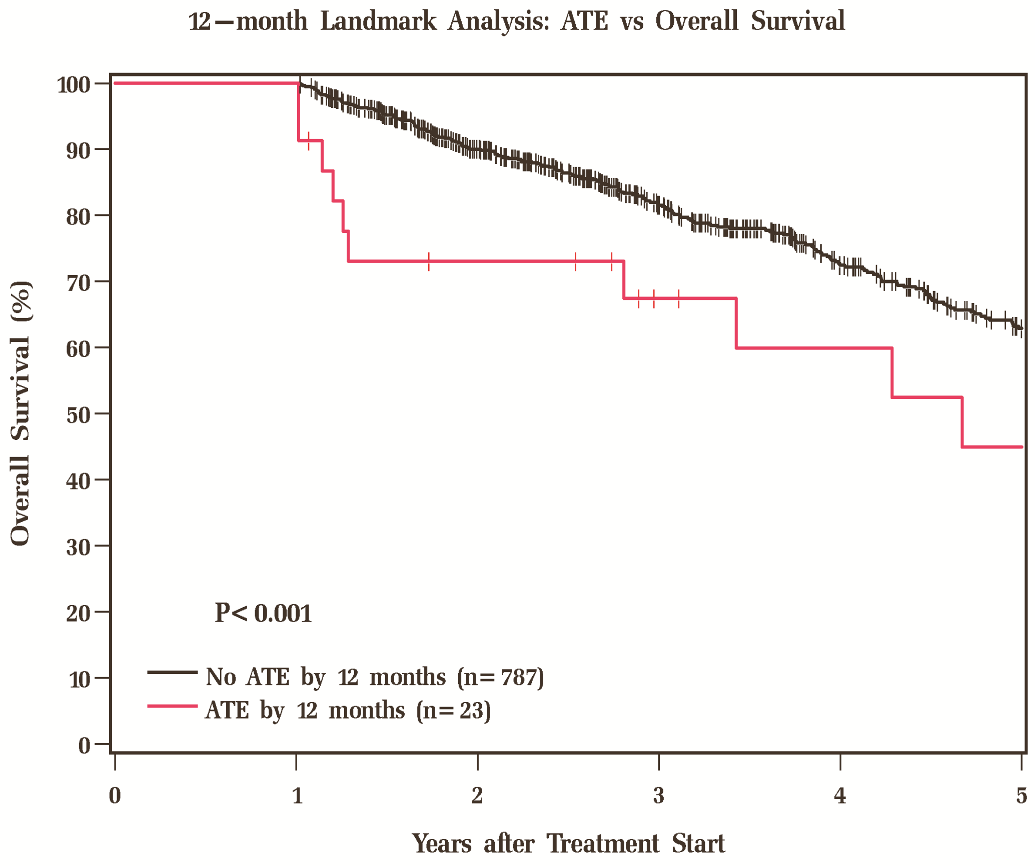

Appendix A. Landmark Analysis for Overall Survival
